# Pain After Lower Limb Amputations: Insights from the Heidelberg Amputation Registry

**DOI:** 10.3390/medicina60111887

**Published:** 2024-11-18

**Authors:** Timo Albert Nees, Cornelia Matt, Julian Deisenhofer, Julia Block, Sebastian I. Wolf, Tobias Renkawitz, Burkhard Lehner, Merkur Alimusaj

**Affiliations:** Department of Orthopaedics, Heidelberg University Hospital, Schlierbacher Landstraße 200a, 69118 Heidelberg, Germanymerkur.alimusaj@med.uni-heidelberg.de (M.A.)

**Keywords:** pain, amputation, phantom limb pain, phantom limb sensation, residual limb pain, stump pain, prosthesis

## Abstract

*Background and Objectives*: The experience of unpleasant sensory phenomena after lower limb amputations (LLAs), including phantom limb pain (PLP), phantom limb sensation (PLS), and residual limb pain (RLP), impacts global healthcare and adversely affects outcomes post-amputation. This study aimed to describe the distribution of PLP, PLS, and RLP among patients with LLAs registered in the Heidelberg Amputation Registry. The primary objective was to determine the prevalence of sensory abnormalities across different amputation levels and causes. *Materials and Methods*: In this single-center, cross-sectional study, data from 459 patients registered in the Heidelberg Amputation Registry were analyzed for the occurrence of PLP, PLS and RLP. Subsequently, logistic regression models were used to identify the independent risk factors associated with sensory disturbances following LLAs. The mean age of the LLA patients (31% female, 69% male) was 58 years (SD ± 18). *Results:* The patients were, on average, 44 years old (SD ± 22) at the time of amputation, with a mean duration since amputation of 15 years (SD ± 17). Transtibial amputations were the most common (43%), followed by transfemoral (39%) and partial foot amputations (10%). Hip and knee disarticulations were observed in 3.7% and 3.5% of the cohort, respectively, with hemipelvectomies accounting for 1%. Traumatic injuries (32%) and neoplastic disorders (22%) were the leading causes of LLAs, while peripheral artery disease and diabetes were responsible for 12% and 6% of cases, respectively. Importantly, a significant proportion of participants (85%) reported experiencing abnormal sensations. The prevalence rates for phantom limb pain (PLP), phantom limb sensation (PLS), and residual limb pain (RLP) were 58%, 66%, and 46%, respectively. The occurrence of sensory disturbances, with the exception of RLP, was significantly affected by the level of amputation. Notably, the age at amputation emerged as an independent risk factor for developing abnormal sensations, including PLS. *Conclusions*: In conclusion, this study provides a comprehensive overview of sensory abnormalities in a diverse cohort of LLA patients, highlighting the age at amputation as an important factor. The findings emphasize the role of comprehensive registries in enhancing care for individuals with amputations and guiding targeted pain management strategies.

## 1. Introduction

Lower limb amputations (LLAs), primarily necessitated by chronic conditions such as diabetes mellitus and peripheral arterial disease, also result from infections, traumatic injuries, and neoplastic disorders. In Germany, the incidence of LLAs is remarkably high, with over 60,000 cases recorded in 2019 [1]. These procedures have a substantial impact on global healthcare and profoundly affect patients’ quality of life. The post-amputation phase is critical, demanding a thorough understanding of the biopsychosocial consequences and factors influencing the outcomes after an amputation. To effectively address these aspects, an interdisciplinary treatment approach synergizing the expertise of surgeons, physiotherapists, occupational therapists, prosthetists, and psychologists is needed. A primary challenge in this context is unpleasant sensory phenomena, including persistent pain post-amputation, which can hinder rehabilitation, including prosthesis fitting, and adversely affect the return to daily activities and optimal quality of life. Therefore, addressing both painful and non-painful sensations post-amputation is a primary focus in clinical research and management strategies.

The experience of unpleasant sensations following LLA is complex, involving various types, such as phantom limb pain (PLP), phantom limb sensation (PLS), and residual limb pain (RLP). PLP is defined as pain perceived in a body region that is no longer present [2]. PLP is the most recognized post-amputation pain syndrome, with painful sensations in the missing portion of the amputated limb often described as cramping, burning, or shooting, particularly in the distal part of the absent limb [3,4]. PLP is typically episodic, varying in duration from seconds to hours. However, a small proportion of patients experience severe and constant PLP. In the literature, the prevalence of PLP varies, but it is estimated to be around 60–80%. The majority of cases are reported to develop within the first week post-amputation [3,4]. The key risk factors for PLP include intense preoperative pain, bilateral amputations, concurrent stump pain, multiple limb surgeries, and advancing age [5]. Although alterations have been described along the entire pain pathway, including in the primary nociceptive afferents, the spinal cord, and the somatosensory cortex, the exact pathophysiological mechanisms underlying PLP remain elusive [5,6,7]. Notably, maladaptive plasticity, such as cortical reorganization, is closely linked to PLP severity [8,9]. Therefore, early-onset interventions to restore the body schema and mitigate reorganizational processes are critical to both preventing and treating PLP [10]. In this context, targeted prosthesis management plays a vital role in redefining the body schema and has been demonstrated to significantly reduce PLP [10,11,12]. Furthermore, the use of a prosthesis not only aids in reconstructing the body image but also in re-establishing sensory feedback from the absent limb, thereby minimizing incongruent sensorimotor functions. This contributes to positive outcomes following PLP treatment [10].

Phantom limb sensation (PLS) comprises non-painful sensory phenomena perceived in the missing body part. PLS encompasses kinetic, proprioceptive, and exteroceptive sensations [13]. Patients experiencing PLS may perceive the length or volume of the absent body part or experience sensations like itching, tingling, and pressure in the amputated area. The prevalence of PLS in individuals with LLAs is notably high, particularly in traumatic amputees, with estimates suggesting up to 80% experience PLS at some point in their lives [14].

Beyond noxious and innocuous sensations in the absent limb, pain in the remaining part of an amputated limb, known as residual limb pain (RLP), represents a chronic complication post-LLA [15]. RLP, resulting from multiple, often overlapping and time-evolving etiologies, encompasses both somatic and neuropathic pain in the stump. Somatic pain etiologies typically include infection, wound complications, unstable scars, osteomyelitis, vascular insufficiency, bone spur formation, hematoma, soft tissue inflammation, and inadequate myoplasty for prosthetic use [5,16,17]. The neuropathic causes primarily involve neuroma or nerve compression and, in some cases, may manifest without a discernible pain generator, resembling complex regional pain syndrome. Systematic overviews with meta-analyses indicate that RLP affects over 50% of LLA patients [16,18].

Despite advancements in surgical techniques, pain management, and prosthesis fitting, pain and phantom sensations continue to pose significant clinical challenges following LLAs. The influence of the amputation level and underlying cause on the development of pain and phantom sensations remains underexplored. Addressing these issues necessitates systematic symptom assessment and an understanding of the factors impacting amputees’ lives. The establishment of amputation registries is crucial to laying a data foundation for answering unresolved questions and enhancing interdisciplinary patient management. The recently established Heidelberg Amputation Registry exemplifies this approach. It comprehensively assesses daily living abilities, comorbidities, locomotor skills, and prosthesis fitting history through self-reported questionnaires and objective evaluations of range of motion, muscle tone, stump condition, and pain conditions. The registry aims to monitor and improve post-LLA treatment and identify factors influencing outcomes. The Heidelberg Amputation Registry is the first in Germany to systematically document the clinical trajectory of patients following LLAs. It is unique for its comprehensive data collection from patients, orthopedic surgeons and technicians. This multidisciplinary approach, aligning with international initiatives like Sweden’s SwedeAmp [19] and the U.S.’s Limb Loss and Preservation Registry [20], ensures continuous monitoring across all the stages of LLA care. Unlike studies using ICD-10 codes [1,21], this registry provides a more detailed and patient-centered approach, allowing for a deeper understanding of clinical outcomes and improvements in care quality.

This study represents the first to report pain characteristics from the Heidelberg Amputation Registry, focusing on a unique cohort with a high proportion of LLAs due to neoplastic disorders and trauma, unlike other registries that primarily include cases of diabetes and vascular disease. Our aims are to (i) describe the distribution of phantom limb pain, phantom limb sensation, and residual limb pain across this diverse population and (ii) evaluate whether these sensations vary based on the cause and level of amputation. Overall, this study also contributes to enhancing the quality of our registry.

## 2. Materials and Methods

### 2.1. Heidelberg Amputation Registry: Data Collection Framework

In the interest of standardizing care for lower limb amputees and due to the variability in treatment strategies across medical specialties, the Heidelberg Amputation Registry was established in 2013. Its primary aim is to create a comprehensive database to document and monitor the continuum of care for individuals with lower limb amputations. Clinical data from patients presenting at our department are prospectively captured. Utilizing a uniform medical and prosthetic documentation system, alongside self-reported patient questionnaires, we collect detailed clinical information as well as several performance-based outcome measures. These methodologies have been elaborated upon in prior publications [22,23]. The assessment is bifurcated into two distinct segments: Part (A) and Part (B). Part (A) entails a patient-centric questionnaire that gathers personal data, encompassing relevant comorbidities, historical and ongoing amputation-related interventions (such as physiotherapy and gait training), prosthetic alignment and utilization, social circumstances, routine activities, and mobility skills. Conversely, Part (B) involves the documentation by an orthopedic specialist, who, on the day of the patient’s evaluation, records specific data pertaining to the amputation. This includes the level and cause of the amputation, the patient’s age at the time of amputation, and the hospital where the amputation was performed. Additionally, this segment includes an assessment of the patient’s range of motion and strength, the condition of the stump (encompassing its shape, skin integrity, and muscle coverage), and the presence of pain. Pain is specifically categorized as residual limb pain (RLP) and phantom limb pain (PLP), and it is documented dichotomously (yes/no), along with phantom limb sensation (PLS). PLP is defined as a painful sensation perceived in the amputated limb, PLS as a non-painful sensation arising from the amputated limb, and RLP as pain originating from the amputated stump. To ensure the consistency and accuracy of the data collection, the clinicians use a uniform documentation system with predefined categories and assessment protocols [22,23]

### 2.2. Study Design and Data Collection

The study population comprised patients with LLAs who presented at our department between August 2013 and December 2019 (cut-off date for analysis: 31 December 2019) and were subsequently registered in the Heidelberg Amputation Registry. The LLAs encompassed a range of amputations: hemipelvectomies (HPs), hip dislocations (HDs), transfemoral (TF) amputations, knee dislocations, and transtibial (TT) and partial foot amputations, including Chopart and Lisfranc joint amputations. Exclusions were made for amputations at the toe level and upper limb amputations.

The amputation causes, as documented in Part (B) of the registry, were categorized according to the primary cause of the amputation: trauma, tumor, peripheral artery disease (PAD), diabetes, infection, and other vascular disorders, with the latter including conditions such as thromboembolic events and aneurysms. The “other” category includes diverse and less common causes of amputation, such as scleroderma, gout, and congenital disorders, which vary widely in etiology. These conditions were grouped together due to their low frequency, making it impractical to analyze them as separate categories. This approach allowed us to capture rare causes that do not fit into the primary categories while still including them in the overall analysis. The examples provided are representative, illustrating the varied etiologies within this group. It is noteworthy that the amputation causes were not always distinctly defined, and instances of crossover or multiple causative factors (e.g., trauma leading to infection, diabetes complicated by infection) were observed. In cases where a clear primary cause could not be determined, multiple contributing factors were documented to capture the complexity of these cases. To this end, a free-text field allows clinicians to briefly describe the trajectory leading to LLA. The sociodemographic and clinical data collected included sex, age, age at amputation, duration since amputation, and body mass index (BMI). These characteristics were utilized for descriptive purposes only and did not influence the selection or exclusion of patients for this study.

This study was conducted in accordance with the Declaration of Helsinki, and it was approved by the Institutional Review Board of the Medical Faculty Heidelberg at Heidelberg University (protocol code S-210/2019, date of approval: 23 April 2019). Written informed consent was obtained from all the subjects involved in the study.

### 2.3. Data Analyses

Data extraction was conducted for a total of 459 patients who met the inclusion criteria for LLAs. The primary focus of the analysis was the assessment of the pain characteristics, specifically RLP, PLP, and PLS. A subgroup categorized as having “abnormal sensations” was created, encompassing patients who reported experiencing at least one of the following: RLP, PLP, or PLS. This grouping was essential for capturing the full spectrum of sensory disturbances, as these sensations often co-occur, overlap, or may be challenging to classify distinctly by the patient. By analyzing them collectively, we aimed to identify broader patterns and associations that may not be apparent when examining each type individually. Descriptive analyses were performed to elucidate the sociodemographic characteristics of the study cohort. This included examining variables such as age, sex, age at amputation, duration since amputation, and body mass index (BMI). The pain characteristics were primarily expressed in terms of their prevalence, operationalized as a dichotomous outcome (yes/no) for each type of pain (RLP, PLP, and PLS). This approach facilitated a clear understanding of the distribution and frequency of the pain types within the study population.

### 2.4. Statistics

Statistical analyses were performed using IBM SPSS Statistics version 26 (IBM, Armonk, NY, USA). Data visualization was performed with GraphPad Prism version 9.5.1 for Windows (GraphPad Software, Boston, MA, USA). The clinical and sociodemographic characteristics were presented as the mean ± standard deviation (SD), with ranges for the minimum and maximum values. The pain prevalence was quantified by the number of patients (n) and their percentages (%). The data distribution was systematically assessed using the D’Agostino–Pearson test, and parametric or non-parametric tests were chosen accordingly. Given that nearly all the demographic data were not normally distributed, the Kruskal–Wallis test was employed for comparing patient characteristics across different groups. To assess the associations between the amputation levels and causes and the pain prevalence, a Pearson Chi-square test was conducted for each variable. Independent two-sided t-tests were used to examine the influence of demographic variables on the pain prevalence. Finally, a binary logistic regression model was generated using previously significant tested variables within the univariate tests to determine the independent risk factors for pain. Patients were excluded only from analyses involving variables with missing data. Statistical significance was set at α = 0.05. The following symbols denoting statistical significance where used: * *p* < 0.05, ** *p* < 0.01, and ns (not significant) for *p*-values greater than 0.05.

## 3. Results

### 3.1. Study Population

Table 1 presents the demographic and clinical profiles of 459 patients with LLAs who were registered in the Heidelberg Amputation Registry as of the analysis cut-off date (31 December 2019). The observed study group was predominantly male (69.3%), as the cohort’s female representation stood at 30.7%. The mean age at data collection was 58.3 years (SD ± 17.9), spanning 5 to 97 years. The average age at amputation was 44 years (SD ± 22.1), with a mean post-amputation duration of 14.7 years (SD ± 19.9), ranging from less than a year to 76 years post-LLA. The total study population (TSP) had a mean BMI of 26.5 kg/m^2^ (SD ± 6.5). From the 459 identified LLA patients, comprehensive data on abnormal sensations, including PLP, PLS, and RLP, were obtained for 383 patients (83.4%). Of these, 327 patients (85.4%) reported abnormal sensations. Specifically, data concerning PLP, PLS, and RLP were available from 360, 351, and 373 patients, with prevalence rates of 58.3%, 66.4%, and 46.1%, respectively. No significant differences in patient characteristics were observed across these subgroups (Supplementary Table S1).

Table 2 delineates the distribution of amputation levels and underlying causes within the study population. In brief, transtibial amputations were most prevalent (43%), followed by transfemoral (39%) and partial foot amputations (10%). Hemipelvectomies were rare, constituting only 1% (*n* = 4) of cases, with hip (*n* = 17) and knee disarticulations (*n* = 16) also being infrequent. Traumatic injury emerged as the leading cause of amputations, accounting for 32% (*n* = 146) of cases, while neoplastic disorders were responsible for 22% (*n* = 100). Over half of the trauma-related LLAs occurred at the transtibial level. Conversely, 60% of the amputations due to malignancies were transfemoral. The majority of hemipelvectomies (75%) and hip disarticulations (65%) were necessitated by neoplastic disorders. Peripheral artery disease and diabetes were less common causes, leading to only 12% and 6% of LLAs, respectively. Comprehensive data are presented in Table 2.

### 3.2. Abnormal Sensations

**Figure 1:**
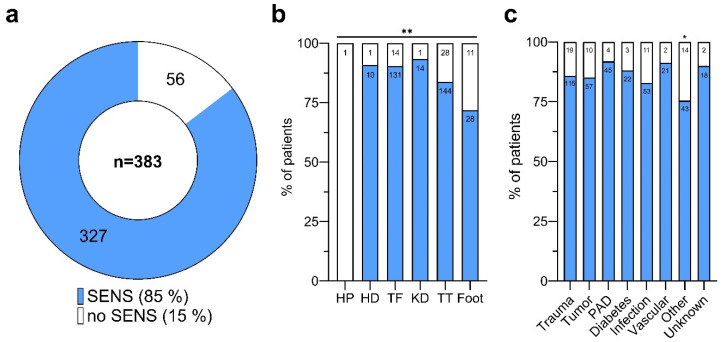
Abnormal sensations after lower limb amputations. (**a**) Distribution of patients reporting abnormal sensations (Sens) post-lower limb amputations. Of those with available data (*n* = 383), a majority (85.4%, *n* = 327) reported experiencing abnormal sensations (blue), while 14.6% (*n* = 56) did not (white). (**b**) Proportion of patients with (blue) and without (white) abnormal sensations, categorized by the amputation level. Asterisks indicate statistical significance (Pearson Chi-square test: ** *p* < 0.01). (**c**) Proportion of patients with (blue) and without (white) abnormal sensations, differentiated by the cause of amputation. Asterisk indicates statistical significance (Pearson Chi-square test: * *p* < 0.05). Patient numbers (n) are shown within the bars. Amputation levels are abbreviated as follows: HP, hemipelvectomy; HD, hip dislocation; TF, transfemoral; KD, knee dislocation; TT, transtibial.

In our cohort, valid data for abnormal sensations post-LLA were obtained from 83% of participants (*n* = 383). Among these individuals, 327 patients (85%) reported experiencing abnormal sensations, whereas only 15% (*n* = 56) did not report PLP, PLS, or RLP, highlighting the high prevalence of both painful and non-painful sensory disturbances after LLAs (Figure 1a). A significant association was observed between the prevalence of abnormal sensations and the level of amputation (Pearson Chi-square test: ** *p* < 0.01; see Supplementary Table S2), with rates varying from 72% in patients with partial foot amputations to 93% in individuals with knee disarticulation (KD) (Figure 1b). The sole patient with HP for whom data were available did not report any sensory abnormalities.

No significant association was detected between the etiology of the amputation (traumatic injuries, malignancies, peripheral artery disease (PAD), diabetes, infections) and the occurrence of abnormal sensations (Figure 1c), with the prevalence of sensory disturbances varying minimally across causes (83% for infections to 92% for PAD). However, the subgroup of patients with unspecific amputation causes (“Other”) was significantly correlated with a lower prevalence of sensory abnormalities (Pearson Chi-square test: * *p* < 0.05; Supplementary Table S3).

Demographic factors such as age, age at amputation, and duration since amputation were found to significantly influence the prevalence of sensory abnormalities post-LLA. In contrast, BMI was not a contributing factor (Table 3).

Further bivariate logistic regression analysis revealed an association between the age at amputation and the prevalence of sensory abnormalities following LLA (*p* = 0.001, Wald coefficient 10.5; Table 4).

### 3.3. Phantom Limb Pain

**Figure 2:**
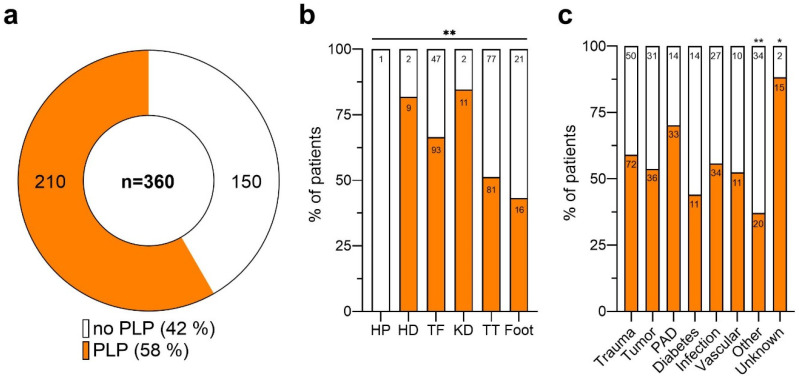
Phantom limb pain after lower limb amputation. (**a**) Distribution of patients reporting phantom limb pain (PLP) post-lower limb amputation. Of those with available data (*n* = 360), 58.3% (*n* = 210) reported experiencing PLP (orange), while 41.7% (*n* = 150) did not (white). (**b**) Proportion of patients with (orange) and without (white) PLP, categorized by the amputation level. Asterisks indicate statistical significance (Pearson Chi-square test: ** *p* < 0.01). (**c**) Proportion of patients with (orange) and without (white) PLP, differentiated by the cause of amputation. Asterisks indicate statistical significance (Pearson Chi-square test: * *p* < 0.05; ** *p* < 0.01). Patient numbers (n) are shown within the bars. Amputation levels are abbreviated as follows: HP, hemipelvectomy; HD, hip dislocation; TF, transfemoral; KD, knee dislocation; TT, transtibial.

For PLP, valid responses were obtained from 360 out of the 459 individuals in the total study population (78%). PLP was reported by 210 patients, representing 58.3% of those surveyed, as depicted in Figure 2a. The remaining 42% (*n* = 150) did not experience PLP. The prevalence of PLP also varied according to the level and cause of the amputation.

Specifically, the prevalence of PLP ranged from 43% in the patients with partial foot amputations to 85% in those with knee disarticulation (KD) (Pearson Chi-square test: ** *p* < 0.01; see Supplementary Table S4). Individuals with TT and TF experienced PLP at rates of 51% and 66%, respectively. Within the HP subgroup, PLP was not reported by the one patient with available data, while the status remained unknown for the other three patients (Figure 2b).

PLP was experienced by 70.2% of patients with PAD-related LLAs. In contrast, 56% of those with diabetes-related LLAs reported no PLP. The occurrence of PLP was 59% in the traumatic injury cases and 54% in the cases related to malignancies. Moreover, 88% of patients with unspecified amputation causes reported PLP, whereas only 37% of patients with various other causes experienced PLP (Figure 2c). A significant association between the cause of the amputation and the prevalence of PLP was only observed for unknown causes (Pearson Chi-square test: * *p* < 0.05) and nonspecific reasons (Pearson Chi-square test: ** *p* < 0.01), as outlined in Supplementary Table S5.

The subsequent logistic regression analysis pinpointed nonspecific causes (“other”) as an independent predictor of lower PLP occurrence (*p* = 0.032, Wald coefficient 4.591; see Table 4). The age at the time of amputation (* *p* < 0.05) and current age (** *p* < 0.01) were significantly associated with the presence of PLP (Table 3), while the duration since amputation and BMI did not show a correlation with PLP occurrence. Nonetheless, these age factors were not associated with the prevalence of PLP in the logistic regression analysis (Table 4).

### 3.4. Phantom Limb Sensation

**Figure 3:**
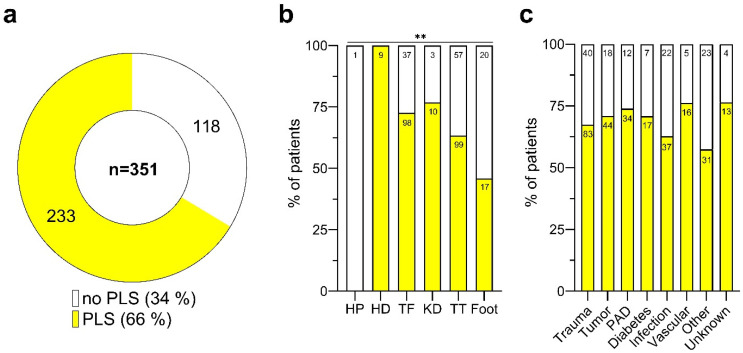
Phantom limb sensation after lower limb amputation. (**a**) Distribution of patients reporting phantom limb sensation (PLS) post-lower limb amputation. Of those with available data (*n* = 351), a majority (66.4%, *n* = 233) reported experiencing PLS (yellow), while 33.6% (*n* = 118) did not (white). (**b**) Proportion of patients with (yellow) and without (white) PLS, categorized by the amputation level. Asterisks indicate statistical significance (Pearson Chi-square test: ** *p* < 0.01). (**c**) Proportion of patients with (yellow) and without (white) PLS, differentiated by the cause of amputation. Patient numbers (n) are shown within the bars. Amputation levels are abbreviated as follows: HP, hemipelvectomy; HD, hip dislocation; TF, transfemoral; KD, knee dislocation; TT, transtibial.

In our cohort, PLS was reported by 233 patients, which constitutes 66% of those with available PLS data (351 out of 459), as shown in Figure 3a, meaning that PLS was not prevalent in the remaining 34%. The prevalence of PLS, like PLP and other sensory abnormalities, was found to be significantly associated with the level of the amputation (Pearson Chi-square test: ** *p* < 0.01; Supplementary Table S6). It was most prevalent among individuals with hip disarticulation (HD), with all nine patients reporting PLS, and least common in patients with partial foot amputation, with a prevalence of 46%. The proportions of PLS in patients with transtibial (TT), transfemoral (TF), and knee disarticulation (KD) amputations were 64%, 73%, and 77%, respectively (Figure 3b).

Similar to PLP, around 70% of patients with PAD-related LLAs experienced PLS. Patients with diabetes and other vascular disorders also demonstrated high rates of PLS (>70%), which contrasts with the prevalence of PLP in these groups (Figure 3c). Additionally, the occurrence of PLS was higher in the trauma and tumor-related LLA cases, at 68% and 71%, respectively, compared to their PLP rates (see Figure 2c). No significant association was found between the cause of the amputation and the presence of PLS (Supplementary Table S7).

Our analysis indicated that the current age, age at amputation, and time since amputation were significantly related to the prevalence of PLS (** *p* < 0.01; Table 3). Further logistic regression analyses revealed associations between the age at the time of amputation and the prevalence of non-painful phantom sensations (*p* = 0.001, Wald coefficient 10.9; see Table 4).

### 3.5. Residual Limb Pain

**Figure 4:**
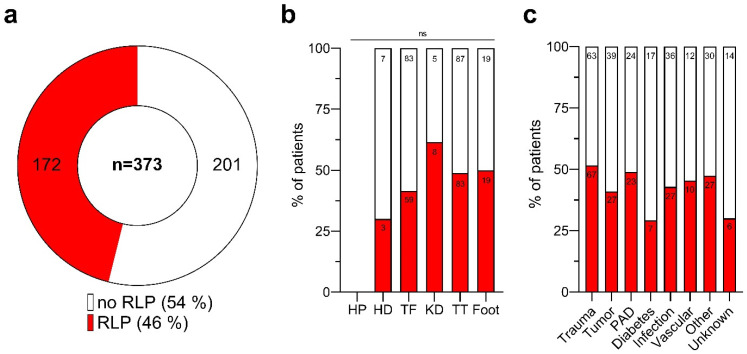
Residual limb pain after lower limb amputations. (**a**) Distribution of patients reporting residual limb pain (RLP) post-lower limb amputation. Of those with available data (*n* = 373), 46.1% (n = 172) reported experiencing RLP (red), while 53.9% (*n* = 201) did not (white). (**b**) Proportion of patients with (red) and without (white) RLP, categorized by the amputation level. Ns, not significant (*p* > 0.05). (**c**) Proportion of patients with (red) and without (white) RLP, differentiated by the cause of amputation. Patient numbers (n) are shown within the bars. Amputation levels are abbreviated as follows: HP, hemipelvectomy; HD, hip dislocation; TF, transfemoral; KD, knee dislocation; TT, transtibial.

Residual limb pain was reported by 172 patients, accounting for 46% of those with available RLP data (*n* = 373) in our study cohort (*n* = 459), as depicted in Figure 4a. Conversely, 53% (*n* = 201) of patients did not experience RLP. The RLP status was unknown for 86 individuals, representing 19% of the total study population. The highest RLP rates were noted following knee disarticulation (KD) at 62%, foot amputations at 50%, and TT amputations at 49%. In comparison, only 30% of HD patients and 42% of TF amputees reported RLP. No data regarding RLP were available for the HP patients within the registry (Figure 4b). Despite these variations, the prevalence of RLP did not significantly correlate with the level of the amputation (Pearson Chi-square test: ns, *p* > 0.05; Supplementary Table S8).

Among the patients with trauma-related LLAs, 52% experienced RLP. Conversely, the majority of patients with diabetes-related amputations (71%) did not report pain at the stump. The proportion of malignancy-related LLA patients experiencing RLP was 41%, akin to the prevalence seen in individuals with infection-related amputations (43%) and those with amputations due to vascular disorders (46%) (Figure 4c).

Interestingly, the cause of the amputation was not a determinant of the prevalence of RLP (Supplementary Table S9). Demographic and amputation factors such as the current age, age at amputation, duration since amputation, and BMI also showed no association with the RLP prevalence (Table 3).

## 4. Discussion

This study offers a comprehensive analysis of the frequency and distribution of common sensory phenomena following lower limb amputation (LLA), including phantom limb pain (PLP), phantom limb sensation (PLS), and residual limb pain (RLP). Drawing on data from 459 patients in the Heidelberg Amputation Registry, this single-center, cross-sectional analysis represents the first exploration of LLA-associated sensory phenomena in this unique cohort. Notably, our cohort deviates from general population trends, with a higher proportion of trauma- and malignancy-related amputations. In contrast to the prevailing causes of major LLAs in Germany 2019—predominantly PAD (51%) and diabetes (19%) [1]—these conditions only accounted for 17% of our study sample. Instead, traumatic injuries (32%) and neoplastic disorders (22%) were the primary causes in over half of our cases. This distribution also differs significantly from data reported in the Healthcare Cost and Utilization Project by Dillingham et al., where trauma and cancer represented only 16% and 1% of cases, respectively [24]. Similarly, the Swedish SwedeAmp registry reports a high prevalence of amputations due to vascular disorders (76%) and diabetes (14%), with trauma comprising just 4% of cases and cancer-related amputations classified under “other” causes [19]. These differences highlight the unique nature of our cohort, which provides critical insights into patient groups that are typically under-represented in the literature. However, compared to population-based studies, data from our single-center registry may have limitations regarding the generalizability of our findings.

In our subgroup with trauma-associated LLAs, encompassing various amputation levels, we observed a prevalence of sensory phenomena ranging from 52% for RLP and 59% for PLP to 68% for PLS. Moreover, 86% of these individuals with trauma-related lower extremity amputations presented any sensory abnormality. This prevalence is consistent with, yet distinct from, previous cross-sectional studies, where sensory abnormality rates after trauma-related LLAs varied, likely due to factors like the amputation level and assessment methodologies [25,26,27]. For instance, the RLP prevalence among U.S. service members and veterans with traumatic amputations, including miscellaneous amputation levels, was reported to be between 48% and 63% [25], and it was as high as 84% in unilateral below-knee amputees from Iran [26]. Esfandiari et al. found a 49% RLP prevalence in a cohort predominantly consisting of trauma-related above-knee amputations (>80%) [27]. Intriguingly, our analysis reveals that traumatic injury, as the causative factor for amputation, does not correlate with an increased likelihood of post-LLA sensory disturbances. Specifically, there was no significant difference in the occurrence of residual limb pain (RLP) or phantom sensations (both noxious and innocuous) in patients with trauma-related amputations compared to the rest of the study cohort.

In our study, the prevalence of sensory disturbances post-LLA due to neoplastic disorders (approximately 85%) was similar to that observed in the trauma subgroup, despite a wider variability in the prevalence rates across different pain types (41% for RLP, 54% for PLP, and 71% for PLS). This observation broadly aligns with the existing literature, although research in this area remains limited. For instance, a prospective study by Ahmed and co-workers involving 160 patients reported PLP and PLS prevalence rates of 41% and 32%, respectively, with RLP being less common at 16% in cancer-related amputations [28]. Our findings also indicated RLP to be the least frequent pain type, with PLP present in about half of the cancer-related amputees. However, our RLP prevalence (41%) was higher compared to Ahmed’s study (16%). A significant discrepancy was observed in the PLS prevalence; our study found PLS in 71% of patients, whereas Ahmed et al. reported it in only one-third [28]. Several factors may explain these discrepancies. Ahmed’s study, conducted in an Indian population, included both upper and lower extremity amputees, whereas our study focused solely on lower limb amputations. Additionally, Ahmed’s study tracked the pain prevalence over time, up to 12 months post-amputation, capturing temporal shifts that may influence pain outcomes. In contrast, the mean time since amputation in our study was 14.7 years, highlighting further differences between these study populations. Demographic differences are also notable. Ahmed’s cohort had a significantly younger mean age (38.2 ± 1.5 years) compared to ours (58.3 ± 17.9 years), likely impacting the pain perception and prevalence. Age plays a crucial role in pain perception following cancer-related amputations, with younger individuals often reporting varied pain experiences. For instance, studies on children and young adults post-cancer-related amputations indicate that while 76% experienced PLP at some point within the first year, the prevalence dropped to just 10% by the end of that year [29], suggesting that pain trajectories may differ significantly by age and over time. Indeed, age has been identified as a significant independent risk factor for developing phantom limb pain (PLP) in a study by Huo and colleagues [30], which analyzed a Chinese population of 168 patients with bone and soft tissue tumor-related amputations. This multivariate logistic regression analysis also highlighted other independent risk factors: preoperative pain, the number of operations, lower limb amputations (as opposed to upper limb amputations), and a more proximal amputation level (above the elbow for upper limbs and above the knee for lower limbs). This finding aligns with previous research [28,31,32] reporting a higher likelihood of PLP in patients with proximal tumor amputations. Independent of the cause of the amputation, a proximal site of amputation has been associated with higher rates of PLP, as evidenced by numerous studies, including a systematic review and meta-analysis [4,28,30,32,33]. In line with this evidence, our study found a higher prevalence of PLP among individuals with above-knee amputations (HD 82% and TF 66%) compared to those with below-knee amputations (TT 51% and partial foot 43%). These observations corroborate findings from previous research. The reasons why a proximal site of amputation poses a higher risk of PLP are not fully understood. Generally, proximal LLAs, such as HD and HP, result in greater surgical trauma, more significant blood loss, and extended recovery periods. These factors elevate the risk of wound complications, infections, and perioperative pain [4,30,34]. As a result, there is increased nerve fiber damage, potentially leading to regenerative processes such as aberrant sprouting of nociceptive fibers and neuroma formation. This, in turn, can cause heightened activity of nociceptors in the periphery and spontaneous activity in the dorsal root ganglia, contributing to the risk of PLP [10,35,36]. Additionally, the prolonged recovery period associated with more proximal amputations may delay the initiation of early therapeutic interventions aimed at preventing PLP. Nonetheless, the timely implementation of both pharmacological and non-pharmacological strategies is essential for mitigating severe PLP [10]. Moreover, proximal amputations involve a larger projected area of the cortex, indicating a more severe disruption of the body scheme, which could further exacerbate PLP [30]. Nonetheless, further experimental studies are required to elucidate these mechanisms fully.

Overall, our study underscores the significant clinical importance of sensory disturbances, including PLP, following LLA. A recent meta-analysis of 39 studies reported an estimated PLP prevalence of 64% among adults post-amputation [4], aligning closely with our finding that 58% of our study cohort experienced PLP. However, our cohort significantly differs from the general population, containing fewer patients with LLAs resulting from peripheral arterial disease (PAD) and diabetes, the leading causes of amputations in Germany. This discrepancy highlights the critical need for effective pain management strategies in the care of individuals with amputations, including both pharmacological and non-pharmacological interventions [5,37,38]. Pharmacological treatment options include antidepressants, anticonvulsants, NMDA antagonists, calcitonin, calcium channel blockers, beta blockers, and local anesthetics, although the evidence for favoring specific options remains limited [38]. Treatment decisions often depend on patient factors, such as comorbidities like depression [38]. We also recognize that the underlying cause of the amputation may influence the therapeutic options; for example, cancer-related amputees may undergo chemotherapy, which could affect their pain types [39,40] and management needs compared to trauma-related cases. Another key component of treatment involves prosthetic replacement of the absent limb, which aids in reconstructing sensory feedback, minimizing incongruent sensorimotor functions, and restoring body image through prosthesis embodiment [10]. Beyond the beneficial effects of prosthesis use on PLP, there is evidence suggesting that the functionality of the prosthesis significantly influences the PLP level. Specifically, patients with upper limb amputations report decreased PLP with functional prostheses compared to cosmetic, non-functional ones [10,11]. Innovative treatments aiming at the restoration of the body scheme and integration of sensorimotor inputs, such as the combination of virtual reality with established mirror therapy, show promising outcomes [41,42,43,44]. Further analysis and correlation of prosthesis usage and pain characteristics from the Heidelberg Amputation Registry could elucidate the effectiveness of these therapeutic approaches in future research.

Interestingly, our study uniquely contributes to the literature by assessing pain and phantom sensations following knee disarticulation (KD), a topic not extensively explored in previous research. We found that KD patients exhibited the highest prevalence of abnormal sensations among all the LLA levels, with 93% reporting sensory disturbances. Specifically, RLP was experienced by 62% of KD patients, while PLP and PLS were reported by 85% and 77% of these patients, respectively. Interestingly, the prevalence of residual limb pain (RLP) was lowest in the group with diabetes, a phenomenon that could be attributed to the presence of diabetic neuropathy. Although our registry did not document specific data on diabetic neuropathy, large epidemiological studies suggest that approximately 50% of all individuals with diabetes develop neuropathy [45], often leading to decreased sensation [46]. This decreased sensitivity may mitigate the perception of RLP in patients with diabetes. Moreover, 46% of the LLA patients in our study cohort reported experiencing RLP, which is in line with previous findings from meta-analyses in which RLP was seen in half of the patients following LLA [16,18]. Compared to PLP and PLS, we could not identify significant associations between RLP prevalence and demographic as well as amputations factors. RLP encompasses a range of etiologies, serving as an umbrella term that includes both somatic and neuropathic pain types within the stump, often with overlapping symptoms [16]. Somatic RLP typically arises from local conditions such as infection, vascular insufficiency, bone spur formation, inadequate wound closure, unstable scarring, and soft tissue inflammation, all of which can hinder effective prosthetic use [16,17,18]. On the other hand, neuropathic RLP is frequently linked to specific underlying issues like neuromas or nerve compression. For example, the prevalence of symptomatic neuroma following LLA was identified to be 15%. Although RLP can manifest without an identifiable pain source, as observed in complex regional pain syndrome cases [16,17], the majority of RLP instances are attributed to localized conditions of the stump rather than demographic factors. Consequently, future research will focus on incorporating stump-related factors from the Heidelberg Amputation Registry to pinpoint the independent risk factors that predict RLP development.

Importantly, our analysis identified the age at the time of amputation as an independent risk factor for developing abnormal sensations, including phantom limb sensation (PLS) and post-lower limb amputation (LLA). This is consistent with the existing literature that suggests an increased prevalence of phantom sensations in patients who are older at the time of LLA [30,32,47]. Notably, our study found that common and specified amputation causes were not independently associated with the development of sensory abnormalities post-LLA. However, the “Others” subgroup, comprising less common causes, including congenital disorders, showed a significant negative association with the phantom limb pain (PLP) prevalence. Interestingly, patients within this category were less likely to experience PLP. The heterogeneity and atypical nature of this subgroup, including the younger age at amputation (congenital causes for LLAs such as dysmelia and congenital pseudarthrosis), might contribute to the lower prevalence of sensory abnormalities. However, due to its diversity, drawing conclusive results is challenging, highlighting a limitation of our study. Furthermore, we did not test for interactions between variables, limiting our ability to detect possible synergistic effects on pain outcomes. While our focus on the main effects helped clarify the independent risk factors, exploring the interactions is a valuable direction for future research.

Beyond our findings, numerous demographic and clinical factors have been shown to impact pain development after amputation, as reviewed by Ishigami et al. [48]. Preoperative risk factors for PLP include nonmodifiable characteristics (such as age, sex, and race/ethnicity) and modifiable ones (including socioeconomic support, severity of chronic conditions, preoperative pain, absence of counseling, psychiatric disorders, and multiple surgeries). Recent research highlights two major preoperative risk factors: an older age and a limited response to prior pain treatments [48,49,50]. Factors related to the amputation procedure, such as advanced techniques like target muscle reinnervation and regenerative peripheral nerve interfaces, have been associated with a reduced incidence of PLP [48,51,52,53]. The post-amputation risk factors include postsurgical pain, shorter residual limb length in lower limb amputations, and the intensity of both PLS and telescoping [48]. Interestingly, the use of prosthetics with a somatosensory biofeedback system has been shown to reduce both the severity and the frequency of PLP [54], and higher rates of prosthesis ownership are associated with lower levels of both PLP and RLP [55]. Additionally, experiencing phantom movement may offer protective effects against PLP [49].

Another limitation stems from the registry-based nature of this study, which relies heavily on the quality of data input and maintenance, as well as the accuracy of the self-reported questionnaires and clinical documentation by orthopedic specialists. While data regarding abnormal sensations were available for 83.4% of our study population, the cause of the amputation remained unclear or uncategorized for a significant portion (19.8%). One explanation could be the unclear association between the primary disease and the secondary reason for amputation—e.g., infection after trauma. The implementation of an expert panel for categorization could potentially address these incongruities in future studies, while a clear convention may improve the definitions. Additionally, incorporating standardized and validated assessment tools (e.g., McGill Pain Questionnaire [56] for pain assessment, the Prosthetic Use Score for prosthesis use [57], and the EQ-5D-5L for quality of life), alongside objective measures like the Timed-Up-and-Go Test [58], neuroimaging [59], wearable technology [60], and novel software solutions [61], could enhance the data consistency and reliability. For instance, implementing innovative tools for standardized documentation of PLS and PLP, including body image quantification, may further address these limitations [61].

Additionally, in our study, data were collected cross-sectionally from 2013 to 2019, without structured follow-up assessments at fixed intervals. This cross-sectional design limits our ability to evaluate the changes in pain over time or to capture the fluctuations in pain that may occur post-amputation. The timing of the assessment is important, as both the duration since amputation and the patient age are known factors that can influence the development of post-amputation pain. This limitation could be addressed in future analyses of this registry through structured follow-up assessments to capture the dynamic changes in pain over time. Future studies might also benefit from including factors like neuroma formation, stump complications, revision surgeries, and pain medications to provide a more comprehensive understanding of the pain dynamics post-LLA. It is important to note that a potential systemic selection bias could affect the data within our registry, given that individuals presenting or referred to our outpatient clinic—and consequently included in our database—are likely to have underlying issues. Therefore, when interpreting the data, it is crucial to recognize that our study cohort might represent a negative selection, indicating that the participants may not be representative of the general population of individuals with amputations.

## 5. Conclusions

In conclusion, our study offers an in-depth overview of the sensory abnormalities in a unique LLA patient cohort and underscores the value of amputation registries in monitoring and standardizing amputee care. It also highlights areas for improvement in terms of data collection and categorization, particularly for patients with unique characteristics in an already diverse population. Nonetheless, this study lays a solid foundation for more detailed future pain research within our registry and emphasizes the importance of continued refinement of the management and understanding of pain in individuals with amputations.

## Figures and Tables

**Table 1:** Study population: patient characteristics.

	TSP	Abnormal Sensations	PLP	PLS	RLP
Patients available, *n (%)* Patients with symptom, *n (%)*	459 (100)	383 (83.4) 327 (85.4)	360 (78.4) 210 (58.3)	351 (76.5) 233 (66.4)	373 (81.3) 172 (46.1)
Sex, *n* (%)					
Male	318 (69.3)	232 (70.9)	146 (69.5)	162 (69.5)	116 (67.4)
Female	141 (30.7)	95 (29.1)	64 (30.5)	71 (30.5)	56 (32.6)
Age, years	58.3 ± 17.9 (5–97)	60.3 ± 16.2 (13–97)	61 ± 14.9 (13–87)	60.7 ± 16.3 (13–97)	59.4 ± 15.8 (13–91)
Age at amputation, years	44.1 ± 22.1 (0–87)	46.8 ± 20.9 (0–87)	47 ± 20.2 (0–83)	48.7 ± 20.7 (6–87)	45.1 ± 20 (0–84)
Time since amputation, years	14.7 ± 16.9 (0–76)	13.97 ± 16.6 (0–76)	13.9 ± 16 (0–74)	12.4 ± 15.9 (0–76)	15.3 ± 17.4 (0–74)
BMI, kg/m^2^	26.5 ± 6.5 (11.5–80.9)	26.5 ± 6.4 (14.2–53.1)	26.5 ± 6.1 (14.5–46.3)	26.3 ± 6.5 (14.2 ± 53.1)	26.3 ± 6.3 (14.2–53.1)

Demographic characteristics of the total study population (TSP) and its subgroups, as categorized based on the presence of abnormal sensations, including phantom limb pain (PLP), phantom limb sensation (PLS), and residual limb pain (RLP). It includes the number (n) and percentage (%) of patients with valid data in each subgroup, as well as the number and proportion (%) of patients experiencing each specific symptom. Data on age, time since amputation, and body mass index (BMI) are expressed as the mean ± standard deviation (range) for each parameter.

**Table 2:** Study population: amputation level and cause.

Amputation Level	TSP	HP	HD	TF	KD	TT	Foot
*n (%)*	459 (100)	4 (0.9)	17 (3.7)	181 (39.4)	16 (3.5)	195 (42.5)	46 (10.0)
* Amputation cause, *n (%)*							
Trauma, *n* % of amputation cause % of amputation level	146 (31.8)	1 0.7 25	0 0 0	44 30.1 24.3	5 3.4 31.3	81 55.5 41.5	15 10.3 32.6
Tumor, *n* % of amputation cause % of amputation level	100 (21.8)	3 3 75	11 11 64.7	60 60 33.1	3 3 18.8	16 16 8.2	7 7 15.2
PAD, *n* % of amputation cause % of amputation level	53 (11.6)	0 0 0	0 0 0	26 49.1 14.4	3 5.7 18.8	18 34 9.2	6 11.3 13.0
Diabetes, *n* % of amputation cause % of amputation level	26 (5.7)	0 0 0	0 0 0	6 23.1 3.3	0 0 0	14 53.8 7.2	6 23.1 13.0
Infection, *n* % of amputation cause % of amputation level	70 (15.3)	0 0 0	3 4.3 17.6	26 37.1 14.4	2 2.9 12.5	30 42.9 15.4	9 12.9 19.6
Vascular, *n* % of amputation cause % of amputation level	26 (5.7)	0 0 0	1 3.8 5.9	12 46.2 6.6	0 0 0	12 46.2 6.2	1 3.8 2.2
Other, *n* % of amputation cause % of amputation level	62 (13.5)	0 0 0	1 1.6 5.9	17 27.4 9.4	3 4.8 18.8	38 61.3 19.5	3 4.8 6.5
Unknown, *n* % of amputation cause % of amputation level	29 (6.3)	0 0 0	2 6.9 11.8	10 34.5 5.5	2 6.9 12.5	13 44.8 6.7	2 6.9 4.3

Clinical characteristics of the total study population (TSP), as categorized by the level of amputation (columns) and the underlying cause (rows). Data are displayed as the number of patients (n) and their corresponding proportions (%). The amputation levels include hemipelvectomy (HP), hip disarticulation (HD), transfemoral (TF), knee disarticulation (KD), and transtibial (TT). * Note: the sum of the patients and percentages categorized by amputation cause exceeds the total number of patients in the TSP and 100%, respectively, due to some patients reporting multiple amputation causes.

**Table 3:** Association between demographic factors and the prevalence of sensory abnormalities post-LLA.

		Unpaired *t*-Test				
Parameter	Pain	*p*-Value	Mean Difference	SD	95% Confidence Interval	
					Lower	Upper
Age	SENS	0.002	9.306	2.955	3.406	15.205
	PLP	0.010	4.961	1.899	1.222	8.700
	PLS	0.005	5.675	1.985	1.763	9.587
	RLP	0.535	1.099	1.770	−2.381	4.580
Age at amputation	SENS	0.000	14.577	3.509	7.564	21.590
	PLP	0.047	5.049	2.526	0.075	10.023
	PLS	0.000	11.535	2.584	6.440	16.631
	RLP	0.711	0.892	2.408	−3.846	5.630
Time since amputation	SENS	0.047	−5.872	2.896	−11.661	−0.084
	PLP	0.509	−1.303	1.968	−5.178	2.573
	PLS	0.006	−5.923	2.115	−10.095	−1.752
	RLP	0.659	0.848	1.918	−2.926	4.622
BMI	SENS	0.890	0.2146	1.5379	−2.8640	3.2932
	PLP	0.899	0.1101	0.8701	−1.6043	1.8245
	PLS	0.600	−0.5034	0.9572	−2.3927	1.3859
	RLP	0.702	−0.3047	0.7962	−1.8710	1.2616

Relationship between the prevalence of sensory abnormalities (SENS), including phantom limb pain (PLP), phantom limb sensation (PLS), and residual limb pain (RLP), and demographic factors (age, age at amputation, time since amputation, and BMI). Significance (exact *p*-value), mean difference, standard deviation (SD), and 95% confidence intervals (lower and upper bounds) of the independent two-sided *t*-tests are shown for each comparison.

**Table 4:** Independent risk factors for pain development post-LLA.

Parameter		B	S.E.	Wald	df	Sig.	OR
**Abnormal Sensation**							
Demographic	Age	0.005	0.010	0.269	1	0.604	1.005
	Age at amputation	0.030	0.009	10.499	1	0.001	1.030
Cause	Other	−0.389	0.423	0.848	1	0.357	0.678
**PLP**							
Demographic	Age	0.003	0.009	0.139	1	0.709	1.003
	Age at amputation	0.012	0.008	2.397	1	0.122	1.012
Cause	Other	−0.716	0.334	4.591	1	0.032	0.489
**PLS**							
Demographic	Age at amputation	0.025	0.008	10.906	1	0.001	1.025
	Time since amputation	−0.001	0.009	0.019	1	0.891	0.999

Outcomes of the bivariate logistic regression analysis aimed at identifying the independent risk factors for pain after lower limb amputation (LLA). The analysis included demographic factors and amputation causes that showed significant associations with the presence of abnormal sensations, phantom limb pain (PLP), and phantom limb sensation (PLS). It was found that age at the time of amputation is a significant independent risk factor for the development of abnormal sensations and PLS. Additionally, amputations due to unspecific causes (labeled as “Other”) demonstrated a lower likelihood of resulting in PLP. Regression coefficients (B), standard errors (S.E.), Wald statistics, degrees of freedom (df), significance levels (Sig.), and the odds ratio (OR) are shown.

## Data Availability

The original contributions presented in this study are included in the article and Supplementary Materials; further inquiries can be directed to the corresponding author.

## Supplementary Materials

The following supporting information can be downloaded at: https://www.mdpi.com/article/10.3390/medicina60111887/s1, Table S1: Data distribution of patient characteristics; Table S2: Abnormal sensations and amputation level; Table S3: Abnormal sensations and amputation cause; Table S4: PLP and amputation level; Table S5: PLP and amputation cause; Table S6: PLS and amputation level; Table S7: PLS and amputation cause; Table S8: RLP and amputation level; Table S9: RLP and amputation cause.
