# Supplementary material for: Pain After Lower Limb Amputations: Insights from the Heidelberg Amputation Registry
Source: Medicina (Kaunas). 2024 Nov 18;60(11):1887. doi: 10.3390/medicina60111887 (PMC11597051; doi:10.3390/medicina60111887)
Supplement: Supplementary file 1 [file medicina-60-01887-s001.zip › medicina-3242377-supplementary.pdf]

Supplementary Data

Supplementary Table S1: Data distribution of patient characteristics

Test for normal distribution (D'Agostino & Pearson test)	Abnormal sensations	PLP	PLS	RLP	Kruskal-Wallis test
**Age**					P-value
K2	10.89	7.591	9.550	3.859	0.6879 (ns)
P value	0.0043	0.0225	0.0084	0.1452	
Passed normality test (alpha=0.05)?	No	No	No	Yes	
P value summary	**	*	**	ns	
**Age at amputation**					
K2	56.30	21.44	46.12	14.03	0.4595 (ns)
P value	<0.0001	<0.0001	<0.0001	0.0009	
Passed normality test (alpha=0.05)?	No	No	No	No	
P value summary	****	****	****	***	
**Time since amputation**					
K2	99.63	69.00	90.97	48.90	0.1254 (ns)
P value	<0.0001	<0.0001	<0.0001	<0.0001	
Passed normality test (alpha=0.05)?	No	No	No	No	
P value summary	****	****	****	****	
**BMI**					
K2	47,36	23,60	35,20	38,01	0.9239 (ns)
P value	<0.0001	<0.0001	<0.0001	<0.0001	
Passed normality test (alpha=0.05)?	No	No	No	No	
P value summary	****	****	****	****	

Supplementary Table S2: Abnormal sensations and amputation level

	**Abnormal sensations**			**Pearson Chi-Square**		
**Amputation level**	No	Yes	Total	Value	df	p-value (asymptotic significance, 2-sided)
Foot	11	28	39	15.877	5	**0.007** **
HD	1	10	11			
HP	1	0	1			
KD	1	14	15			
TF	14	131	145			
TT	28	144	172			
Total	56	327	383			

Supplementary Table S3: Abnormal sensations and amputation cause

		**Abnormal sensations**			**Pearson Chi-Square**		
**Amputation cause**		No	Yes	Total	Value	df	p-value (asymptotic significance, 2-sided)
Trauma	No	37	212	249	0.032	1	0.857
	Yes	19	115	134			
PAD	No	52	282	334	1.877	1	0.171
	Yes	4	45	49			
Tumor	No	46	270	316	0.006	1	0.938
	Yes	10	57	67			
Diabetes	No	53	305	358	0.147	1	0.701
	Yes	3	22	25			
Infection	No	45	274	319	0.405	1	0.524
	Yes	11	53	64			
Vascular	No	54	306	360	0.688	1	0.407
	Yes	2	21	23			
Unknown	No	54	309	363	0.361	1	0.548
	Yes	2	18	20			
Other	No	42	284	326	5.300	1	**0.021***
	Yes	14	43	57			

Supplementary Table S4: PLP and amputation level

	PLP			Pearson Chi-Square		
**Amputation level**	No	Yes	Total	Value	df	p-value (asymptotic significance, 2-sided)
Foot	21	16	37	18.079	5	**0.003****
HD	2	9	11			
HP	1	0	1			
KD	2	11	13			
TF	47	93	140			
TT	77	81	158			
Total	150	210	360			

Supplementary Table S5: PLP and amputation cause

		PLP			Pearson Chi-Square		
**Amputation cause**		No	Yes	Total	Value	df	p-value (asymptotic significance, 2-sided)
Trauma	No	100	138	238	0.035	1	0.851
	Yes	50	72	122			
PAD	No	136	177	313	3.139	1	0.076
	Yes	14	33	47			
Tumor	No	119	174	293	0.717	1	0.397
	Yes	31	36	67			
Diabetes	No	136	199	335	2.271	1	0.132
	Yes	14	11	25			
Infection	No	123	176	299	0.204	1	0.652
	Yes	27	34	61			
Vascular	No	140	199	339	0.325	1	0.569
	Yes	10	11	21			
Unknown	No	148	195	343	6.564	1	**0.010***
	Yes	2	15	17			
Other	No	116	190	306	11.854	1	**0.001***
	Yes	34	20	54			

Supplementary Table S6: PLS and amputation level

	PLS			Pearson Chi-Square		
**Amputation level**	No	Yes	Total	Value	df	p-value (asymptotic significance, 2-sided)
Foot	20	17	37	17.034	5	**0.004****
HD	0	9	9			
HP	1	0	1			
KD	3	10	13			
TF	37	98	135			
TT	57	99	156			
Total	118	233	351			

Supplementary Table S7: PLS and amputation cause

		PLS			Pearson Chi-Square		
**Amputation cause**		No	Yes	Total	Value	df	p-value (asymptotic significance, 2-sided)
Trauma	No	78	150	228	0.102	1	0.749
	Yes	40	83	123			
PAD	No	106	199	305	1.345	1	0.246
	Yes	12	34	46			
Tumor	No	100	189	289	0.710	1	0.400
	Yes	18	44	62			
Diabetes	No	111	216	327	0.229	1	0.632
	Yes	7	17	24			
Infection	No	96	196	292	0.428	1	0.513
	Yes	22	37	59			
Vascular	No	113	217	330	0.963	1	0.326
	Yes	5	16	21			
Unknown	No	114	220	334	0.815	1	0.367
	Yes	4	13	17			
Other	No	0	95	202	2.303	1	0.129
	Yes	1	23	31			

Supplementary Table S8: RLP and amputation level

	RLP			Pearson Chi-Square		
**Amputation level**	No	Yes	Total	Value	df	p-value (asymptotic significance, 2-sided)
Foot	19	19	38	4.214	4	0.378
HD	7	3	10			
KD	5	8	13			
TF	83	59	142			
TT	87	83	170			
Total	201	172	373			

Supplementary Table S9: RLP and amputation cause

		PLS			Pearson Chi-Square		
**Amputation cause**		No	Yes	Total	Value	df	p-value (asymptotic significance, 2-sided)
Trauma	No	138	105	243	2.364	1	0.124
	Yes	63	67	130			
PAD	No	177	149	326	0.173[a]	1	0.678
	Yes	24	23	47			
Tumor	No	162	145	307	0.874	1	0.350
	Yes	39	27	66			
Diabetes	No	184	165	349	2.964	1	0.085
	Yes	17	7	24			
Infection	No	165	145	310	0.323	1	0.570
	Yes	36	27	63			
Vascular	No	189	162	351	0.004[a]	1	0.949
	Yes	12	10	22			
Unknown	No	187	166	353	2.208[a]	1	0.137
	Yes	14	6	20			
Other	No	171	145	316	0.043[a]	1	0.836
	Yes	30	27	57			
